# Disparities in contralateral prophylactic mastectomy use among women with early-stage breast cancer

**DOI:** 10.1038/s41523-017-0004-z

**Published:** 2017-01-27

**Authors:** Younji Kim, Anne Marie McCarthy, Mirar Bristol, Katrina Armstrong

**Affiliations:** 1 Massachusetts General Hospital, 50 Staniford Street, 9-940L, Boston, MA 02114 USA; 2 Harvard Medical School, Boston, MA USA

## Abstract

Contralateral prophylactic mastectomy use has increased over the past decades among women with early-stage breast cancer. Racial differences in contralateral prophylactic mastectomy use are well described, but with unclear causes. This study examined contralateral prophylactic mastectomy use among black and white women and the contribution of differences in perceived risk to differences in use. We surveyed women diagnosed with early-stage unilateral breast cancer between ages 41–64 in Pennsylvania and Florida between 2007–2009 to collect data on breast cancer treatment, family history, education, income, insurance, and perceived risk. Clinical factors—age,stage at diagnosis, receptor status—were obtained from cancer registries. The relationships between patient factors and contralateral prophylactic mastectomy were assessed using logistic regression. The interaction between race and contralateral prophylactic mastectomy on the perceived risk of second breast cancers was tested using linear regression. Of 2182 study participants, 18% of whites underwent contralateral prophylactic mastectomy compared with 10% of blacks (*p* < 0.001). The racial difference remained after adjustment for clinical factors and family history (odds ratio = 2.32, 95% confidence interval 1.76–3.06, *p* < 0.001). The association between contralateral prophylactic mastectomy and a reduction in the perceived risk of second breast cancers was significantly smaller for blacks than whites. Blacks were less likely than whites to undergo contralateral prophylactic mastectomy even after adjustment for clinical factors. This racial difference in use may relate to the smaller impact of contralateral prophylactic mastectomy on the perceived risk of second breast cancers among blacks than among whites. Future research is needed to understand the overall impact of perceived risk on decisions about contralateral prophylactic mastectomy and how that may explain racial differences in use.

## Mastectomy: White women more likely to choose double breast removal

Racial disparities exist in the desire of American women with early-stage breast cancer to surgically remove their healthy breast tissue. Younji Kim and colleagues from the Massachusetts General Hospital in Boston, USA, surveyed more than 2100 middle-aged women from Pennsylvania and Florida who were newly diagnosed with early-stage cancer in one breast. They asked about the women’s medical and family histories, perceived risk of further disease, and assorted demographic variables. After adjusting for clinical factors and family history, white women were more than twice as likely to undergo contralateral prophylactic mastectomy, in which both breasts are surgically removed, than were black women. The authors surmise that the racial discrepancy may result from perceptual differences among populations about the benefits of bilateral mastectomies on future cancer risk, although more research is needed into the decision-making process.

## Introduction

The use of contralateral prophylactic mastectomy (CPM) has increased over the past two decades among women diagnosed with early-stage unilateral breast cancer.^1–6^ Several studies showed that among women who were surgically treated for their unilateral breast cancer, the proportion of women who underwent CPM has increased in recent years.^3,4^ For example, based on Surveillance Epidemiology, and End Results registry data, the proportion of breast cancer patients who underwent CPM increased from 3.9% in 2002 to 12.7% in 2012, however, there was no improvement in long-term survival despite this trend.^4^ CPM is recommended for women with a germline *BRCA1/2* mutation^7^ or a strong family history even without a known mutation since their risk of contralateral breast cancer is higher than other breast cancer survivors.^8–10^ However, for breast cancer patients without *BRCA1/2* mutations or strong family histories, the risk of contralateral breast cancer is relatively low and has declined over time.^9,11,12^ Furthermore, whether CPM does or does not have significant survival benefits is still controversial.^4,5,13–16^

Uptake of CPM has been demonstrated to be substantially higher among white than black women,^1–3,6,17^ with one recent study finding that 10% of black and 20% of white women underwent CPM after a diagnosis of hormone receptor positive cancer.^6^ However, very little is known about the factors that drive racial differences in use of CPM. One factor that may contribute to racial differences in CPM use is differences in perceived breast cancer risk between black and white women. Several studies have found that black women tend to have a lower perceived risk of breast cancer than white women.^18–20^ Furthermore, one study also found that black women had a lower perception of the benefit of screening mammography on breast cancer mortality than white women,^21^ raising the possibility of differences in the perceived effectiveness of other preventive interventions such as CPM. However, to our knowledge, there are no published studies investigating the potential contribution of differences in risk perception to racial differences in use of CPM.

Given this background, this current study examined CPM use among a large, population-based sample of black and white women diagnosed with early-stage breast cancer. In addition to testing the contribution of clinical and socioeconomic characteristics to racial differences in CPM use, we investigated whether the association between CPM use and perceived risk of a second breast cancer differed between black and white women and may have contributed to differences in cancer treatment use.

## Results

Of the 2182 study participants, 1333 (61%) were white and 849 (39%) were black (Table 1). Only a small proportion of participants reported Hispanic ethnicity (2% of black and 7% of white women).The age distribution differed with white women more likely to be in an older age group (*p* = 0.001). Black women were less likely to have any family history of breast or ovarian cancer compared to white women (52% vs. 47%, *p* < 0.001), but a greater proportion of black women had three or more first or second-degree relatives with breast or ovarian cancer compared to white women (24% vs. 20%, *p* < 0.001). Black women were more likely to have negative estrogen receptor (ER)/ progesterone receptor (PR) status, income lower than $30,000 per year, government supported insurance—Medicaid or Medicare—and lower educational attainment than white women. There were also small but statistically significant differences in *BRCA1/2* mutation risk between black and white women, with more white women being at high risk, whereas more black women being at low risk (*p* = 0.04).

**Table1:** Characteristics of study participants by race (*N* = 2182)

	Black		White		
	*N*	%	*N*	%	*p*-value
Total state	849	38.9	1333	61.1	**—**
Florida	475	55.9	749	56.2	0.91
Pennsylvania	374	44.1	584	43.8	**—**
Age					
41–44	86	10.1	122	9.2	0.001
45–49	181	21.3	247	18.5	**—**
50–55	192	22.6	277	20.8	**—**
55–59	220	25.9	307	23.0	**—**
60–64	170	20.0	380	28.5	**—**
Ethnicity					
Hispanic	16	1.9	83	6.2	<0.001
Non-hispanic/Unknown	833	98.1	1250	93.8	**—**
Stage					
I	429	50.5	810	60.8	<0.001
II	420	49.5	523	39.2	**—**
ER/PR					
Negative	253	29.8	201	15.1	<0.001
Positive	547	64.4	1040	78.0	**—**
Unknown	49	5.8	92	6.9	**—**
Education					
≤High school	302	35.6	384	28.8	<0.001
Any college	420	49.5	657	49.3	**—**
Graduate school	112	13.2	279	20.9	**—**
Unknown	15	1.8	13	1.0	**—**
Income					
<30 K	348	41.0	279	20.9	<0.001
30–70 K	268	31.6	436	32.7	**—**
>70 K	139	16.4	535	40.1	**—**
Unknown	94	11.1	83	6.2	**—**
Insurance					
Employer based	316	37.2	660	49.5	<0.001
Medicaid	103	12.1	54	4.1	**—**
Medicare	144	17.0	223	16.7	**—**
Self-pay	145	17.1	290	21.8	**—**
Other/Unknown	141	16.6	106	8.0	**—**
Family member(s) with breast or ovarian cancer					
None	442	52.1	629	47.2	<0.001
1	133	15.7	276	20.7	**—**
2	74	8.7	166	12.5	**—**
3 or more	200	23.6	262	19.7	**—**
BRCA1/2 mutation risk					
High	183	21.6	337	25.3	0.04
Moderate	359	42.3	577	43.3	**—**
Low	307	36.2	419	31.4	**—**

*ER* estrogen receptor, *PR* progesterone receptor

Black women were less likely to undergo CPM compared to white women (10% vs. 18%, *p* < 0.001). When adjusted for age and stage at diagnosis, ER/PR status, and family history of breast or ovarian cancer, white women had more than twice the odds of undergoing CPM compared to black women (Table 2, Model 2, odds ratio = 2.32, 95% confidence interval (CI) 1.76–3.06, *p* < 0.001). In addition, women who were diagnosed with breast cancer at younger ages or at stage II (vs. stage I) or had more family members with breast or ovarian cancer were more likely to undergo CPM (Table 2, Model 2).

**Table 2:** Logistic regression models for contralateral prophylactic mastectomy (*N* = 2182)

	Model 1 Unadjusted model			Model 2 Adjusted for age and stage at diagnosis, ER/PR status, and family history		
	OR	95% CI	*p*-value	OR	95% CI	*p*-value
White vs. black	1.99	1.53–2.60	<0.001	2.32	1.76–3.06	<0.001
Age (vs. 60 and older)						
41–44	**—**	**—**	**—**	4.31	2.77–6.68	<0.001
45–49	**—**	**—**	**—**	2.50	1.69–3.70	<0.001
50–54	**—**	**—**	**—**	1.91	1.28–2.84	0.001
55–59	**—**	**—**	**—**	1.69	1.14–2.51	0.009
Stage						
I	**—**	**—**	**—**	Ref	**—**	**—**
II	**—**	**—**	**—**	1.39	1.09–1.78	0.009
ER/PR						
No	**—**	**—**	**—**	Ref	**—**	**—**
Yes	**—**	**—**	**—**	0.84	0.62–1.14	0.26
Unknown	**—**	**—**	**—**	0.73	0.42–1.27	0.26
Family members with breast or ovarian cancer						
None	**—**	**—**	**—**	Ref	**—**	**—**
1	**—**	**—**	**—**	1.35	0.96–1.89	0.09
2	**—**	**—**	**—**	1.59	1.07–2.35	0.02
3 or more	**—**	**—**	**—**	2.51	1.86–3.39	<0.001

*CI* confidence interval, *ER* estrogen receptor, *OR* odds ratio, *PR* progesterone receptor

As expected, women who had undergone CPM had a substantially lower perceived risk of a second cancer than women who had not undergone CPM (Table 3). Among women who underwent CPM, 39% of whites and 56% of black women reported 0% perceived risk of a second breast cancer (*p* = 0.02), whereas among women who had not undergone CPM, 16% of whites and 41% of black women reported 0% perceived risks of a second breast cancer (*p* < 0.001). However, in an adjusted model, the difference in perceived risk of a second breast cancer between women who had and had not undergone CPM was smaller among black women than among white women (Table 4, *p* = 0.03 for interaction). Similarly, in stratified linear regression, use of CPM was associated with a five point difference in risk perception among black women [*β* = −5.34, 95% CI (−13.12) – (2.44), *p* = 0.18] but a 15 point difference among white women [*β* = −15.28, 95% CI (−19.57) – (−10.99), *p* < 0.001].

**Table 3:** Comparison of patient risk perception of a second breast cancer by race (*N* = 1810)

	White^a^		Black		
	*N*	%	*N*	%	*p*-value
Overall (excluded missing, *n* = 372)					
	**1122**		**688**		
0%	230	20.5	294	42.7	<0.001
1–49%	447	39.8	187	27.2	—
50%	293	26.1	128	18.6	—
51–99%	115	10.2	44	6.4	—
100%	37	3.3	35	5.1	—
Among women who had not undergone CPM (excluded missing, *n* *= *310)					
	**921**		**624**		
0%	151	16.4	258	41.3	<0.001
1–49%	364	39.5	174	27.9	—
50%	269	29.2	122	19.6	—
51–99%	105	11.4	38	6.1	—
100%	32	3.5	32	5.1	—
Among women undergone CPM (excluded missing, *n* = 62)					
	**201**		**64**		
0%	79	39.3	36	56.3	0.02
1–49%	83	41.3	13	20.3	—
50%	24	11.9	6	9.4	—
51–99%	10	5.0	6	9.4	—
100%	5	2.5	3	4.7	—

*CPM* contralateral prophylactic mastectomy

^a^Risk perception of a second breast cancer was measured as a continuous variable through a single survey question: “What do you think your chance is of developing a second breast cancer in your lifetime? Please choose a number between 0% (no chance of breast cancer) and 100% (definitely will get breast cancer).” The responses were categorized into five groups: 0%, 1–49%, 50%, 51–99%, and 100%, with 0% representing women who perceived themselves having no chance of developing a second breast cancer while 100% for women believed that they are likely to develop a second breast cancer

^b^Bold values indicate the total sample numbers stratified by race for each group that is being analyzed (overall, women who had not undergone CPM, or among women undergone CPM)

**Table 4:** Linear regression models for patient risk perception of a second breast cancer by race (*N* = 1810)

	Black					White				
	Coefficient	95% CI			*p*-value	Coefficient	95% CI			*p*-value
CPM^a^	−5.34	−13.12	–	2.44	.18	−15.28	−19.57	–	−10.99	<0.001
Age at diagnosis	0.04	−0.31	–	0.39	.81	−0.11	−0.36	–	0.14	0.39
Stage II (vs. stage I)	−1.39	−5.92	–	3.14	.55	−1.34	−4.61	–	2.06	0.45
ER/PR (vs. negative)										
Positive	−7.57	−12.56	–	−2.59	0.003	−3.83	−8.88	–	0.38	0.07
Unknown	−2.69	−12.32	–	6.95	0.58	2.62	−5.42	–	10.02	0.56

*CPM* contralateral prophylactic mastectomy, *ER* estrogen receptor, *PR* progesterone receptor

^a^ *P* interaction between contralateral prophylactic mastectomy and race = 0.03

## Discussion

The use of CPM among women with early-stage breast cancer has increased substantially in the U.S. over the last decade,^1–6^ with higher rates among white women than black women.^1–3,6,17^ Prior studies have been unable to explain the difference in CPM use by racial differences in clinical or sociodemographic factors. Our findings support this racial difference in utilization and suggest one possible explanation for the difference.

Among the over 2000 women with early stage breast cancer in this current study, black women who did not undergo CPM had a lower perceived risk of a second breast cancer and the apparent impact of CPM on the perceived risk of a second breast cancer was smaller among black than white women, suggesting that black women may have a lower perceived risk of a second breast cancer than white women and that they may see CPM as less effective at lowering that risk. First, the racial differences in perceived risk are supported by findings from other retrospective studies which suggest that, in general, white women are more likely to overestimate risk of developing breast cancer, and perceived risk is lower among black women.^20,22–26^ In addition, several studies have found that the degree of cancer worry of recurrence and perceived risk for contralateral breast cancer can influence decisions about cancer treatments.^9,23,27–30^ A previous study of breast cancer patients diagnosed at age 40 or younger found that those who had undergone CPM had lower degree of worry about recurrence following surgery than women who had not undergone CPM,^23^ which is similar to the pattern of perceived risk observed among white women in our study. In this retrospective study by Rosenberg et al.,^23^ however, 86% of the study population was non-Hispanic white and racial differences in patients’ worry about cancer recurrence were not assessed. Parker et al.^27^ surveyed newly diagnosed early-stage breast cancer patients prior to surgery, and found that women with greater cancer worry were more likely to undergo CPM, though the study did not evaluate differences by race due to a relatively small sample size (<200) and few non-white participants. Among women diagnosed with breast cancer, one study found that black women were more likely than white women to think that the chances of breast cancer recurrence are the same for mastectomy as for other less aggressive treatments.^31^ A second study found that black breast cancer patients reported less worry about cancer returning to the same breast, to the other breast, or spreading to other parts of the body compared with white breast cancer patients.^32^ In addition, a population-based survey of breast cancer patients that included nearly 450 black women found that patients who reported that concerns about breast cancer recurrence were very important in their surgical decision making were more likely to have had mastectomy.^33^ Furthermore, as noted previously, black women without breast cancer have been demonstrated to have a lower perceived risk of developing breast cancer than white women,^20^ even among women with higher than average risk due to family history^18^ or other risk factors.^19^ While these findings suggest a mechanism linking differences in perceived risk to racial differences in CPM, we were not able to measure risk perception prior to CPM use and additional prospective longitudinal studies of risk perception pre and post CPM are needed.

Given that CPM is not currently recommended by current advisory groups for women not known to have *BRCA1/2* mutations,^7,34^ there is no optimal rate of uptake and racial differences may represent overuse among white women as much as underuse among black women. Risk of a contralateral breast cancer is associated with genetic susceptibility, such as germline *BRCA1/2* mutation, or a strong family history of breast cancer and the decision to undergo CPM was associated with family history and cancer stage in our study, suggesting that uptake is partly related to risk factors. However, given that black women are actually at an elevated risk of contralateral and second breast cancers compared to white women,^12,35^ the lower rates of CPM use among black women are surprising if the decision is driven by the risk of recurrence. Although racial differences in preference sensitive decisions can be more complex to unravel, they are an increasingly important source of differences in outcomes in the era of precision medicine. Ensuring that these decisions are informed choices without constraints created by inadequate access or information will be critical for the benefits of precision medicine to reach all populations.

The reasons for these racial differences in risk perception are not clear but this study indicated that the lower risk perception is unlikely to be driven by actual differences in risk. At the same time, minority patients have been found to experience different, and often inferior, communication with their physician and decision-making processes in several areas including cancer treatment.^33,36,37^ Such differences may mean that black women are less likely to receive adequate information about their cancer management options including the risk of a second cancer and the potential impact of CPM on that risk, which may in turn lead to racial differences in treatment use.^38,39^ Furthermore, it is not clear from these results whether white women are more likely to have an exaggerated perception of their second breast cancer risk and overuse CPM or whether black women underestimate their risk and underuse CPM.

This study has several strengths. To our knowledge, it is one of few studies to examine the likelihood of undergoing CPM by race and by patients’ risk perception of second breast cancers in a large, population-based sample of racially diverse breast cancer patients. Also by including both patient survey and cancer registry data, it included a wide range of potential explanations for the disparity in treatment use including age and stage at diagnosis and family history information, which is a critical factor, as well as patients’ attitudes and perceptions of cancer treatments. However, this study has a few limitations. Our patient survey had insufficient evidence to determine whether the racial disparities in CPM use are driven by overuse of the procedure by white women or underuse by black women. Also, we did not adjust for income or insurance status, which might partially underlie racial differences. Additionally, since the patient survey was conducted following breast cancer treatment, we do not know whether risk perception was similar or changed before and after CPM. Risk perception measured prior to the procedure could have enhanced the analysis and allowed assessment of whether differences in risk perception before CPM explained racial differences in CPM use.

Racial disparities in CPM among white and black women are large and may be related to differences in perceived risk of a second cancer and the impact of CPM on that risk. Future studies are needed to determine the underlying causes of differences in perceived risk between black and white women, how perceived risk changes before and after a surgical procedure, and how it impacts their decision-making process for cancer treatments.

## Methods

### Study population and data collection

This study included women who were diagnosed with early-stage unilateral breast cancer at ages 41–64 between January 1, 2007 and December 31, 2009 identified through Pennsylvania and Florida cancer registries. These states were included because of the size and diversity of their populations and the ability to directly contact patients from cancer registry files. Based upon the cancer registry data, the majority of the study participants were residents living in Pennsylvania and Florida with a small portion from neighboring states. We included all black women and an equal random sample of white womento facilitate comparisons by race. Women were sampled based upon race recorded in the cancer registry. Thirty women were excluded because they reported a different race when surveyed. Women were surveyed by mail 2–3 years after cancer diagnosis with additional telephone recruitment of black non-responders up to 4 years from diagnosis. Details of the survey methods have been published.^40^ The survey consisted of a total of 63 questions asking study participants about characteristics of breast cancer, any surgical treatment done for their breast cancer, perceived risk of a second breast cancer, other personal health history and family histories of ovarian or breast cancer. For treatment, women were instructed to report only treatments given for the breast cancer at the time it was first diagnosed, and not include treatments for any cancer that came back after the original treatment. Perceived risk of developing a second breast cancer was measured using the following survey item: “What do you think your chance is of developing a second breast cancer in your lifetime? Please choose a number between 0% (no chance of breast cancer) and 100% (definitely will get breast cancer).” The study was approved by the University of Pennsylvania and Partners Healthcare Institutional Review Boards.

The overall response rate was 61% with the response rate being slightly higher among white women (62%) in comparison to black women (58%) when calculated using the Response Rate Definition #4 defined by the American Association for Public Opinion Research.^41^ Breast cancer treatment use was measured with survey questions asking whether a study participant received following treatments: lumpectomy, mastectomy, bilateral mastectomy, and adjuvant treatments such as radiation therapy, chemotherapy or hormonal therapy. Family history—both first and second degree—of breast or ovarian cancer, education, income, and insurance status were also ascertained through the survey. Age and stage at diagnosis, and ER/PR status were obtained from cancer registry data. At the end of the recruitment, we excluded seven women, who were diagnosed with bilateral breast cancer, based on cancer registry data.

### Statistical analyses

Patient characteristics, including age at diagnosis (5-year categories), stage at diagnosis (AJCC Stages I or II) and ER/PR status (negative, positive, unknown), family history of breast or ovarian cancer in both first and second degree relatives (4 categories: none, 1, 2, and 3 or more affected family members), and *BRCA1/2* mutation risk (categorized high, moderate, and low using age at diagnosis, family history, and Ashkenazi Jewish heritage)^40^ were compared for black and white participants using Chi-Square tests. The effect of patient factors and race on CPM was assessed with logistic regression models adjusted for the factors described above. Perceived risk of a second breast cancer and risk perception stratified by use of CPM were compared between black and white participants using Chi-square tests. Linear regression was used to test the interaction of CPM and race on perceived risk of a second breast cancer, adjusting for age, stage at diagnosis, ER/PR status, and family history of breast and ovarian cancer, and linear regression of the association between CPM and risk perception was stratified by race. All statistical tests were two-sided with a significance level of *p* < 0.05. Statistical software STATA/IC version 14 was used for all analyses (College Station, TX).

### Availability of data and materials

Study subjects did not provide informed consent for public sharing of the study data.
